# Modified oral food challenge protocol approach in the diagnosis of Food Protein-Induced Enterocolitis Syndrome

**DOI:** 10.1186/s13223-022-00651-9

**Published:** 2022-01-31

**Authors:** Jessica Sultafa, Lundy McKibbon, Hannah Roberts, Jumana Sarraj, Harold Kim

**Affiliations:** 1 Department of Medicine, Western University, London, ON Canada; 2 Division of Clinical Immunology and Allergy, Department of Medicine, Western University, London, ON Canada; 3 Division of Clinical Immunology and Allergy, Department of Medicine, McMaster University, Hamilton, ON Canada

**Keywords:** Food Protein-Induced Enterocolitis Syndrome, FPIES, Oral food challenge, Food allergy

## Abstract

**Background:**

Food Protein-Induced Enterocolitis Syndrome (FPIES) is a non-IgE mediated food allergy most commonly presenting in infants. The most common food triggers include soy, cow’s milk and grains. Symptoms may include intractable vomiting, diarrhea, lethargy, pallor, abdominal distention, hypotension and/or shock. Oral food challenges (OFCs) given at food protein dose of 0.06–0.6 g/kg in 3 equivalent doses administered over a few hours are recommended in guidelines to confirm a diagnosis.

**Case presentation:**

The patient is a 6-month-old girl with a history of severe FPIES symptoms to egg. In our clinic, we perform OFC with 1/100 serving dose on visit 1 and then increase the dose monthly. The patient takes the tolerated dose daily at home between visits. An OFC to baked egg at 1/100 of a serving was performed and was well-tolerated on her initial visit. The patient remained on the same dose upon returning home. Within 1-week, she developed FPIES symptoms including watery diarrhea and severe emesis requiring ondansetron. She required an Emergency Department visit for one of the reactions.

**Conclusions:**

Our patient had severe FPIES symptoms with a small amount of egg. We believe that administration of three large food challenge doses on one clinic visit, as guidelines currently suggest, does not allow adequate time for symptoms to appear. Our patient likely would have suffered a severe reaction. Also, this guidelines protocol does not allow for monitoring of more delayed or chronic FPIES. We propose a modified protocol to OFCs with cautious up-dosing to allow for safer OFCs and monitoring of chronic FPIES. We have implemented an OFC approach where only one food challenge dose (starting with 1/100 of final dose) is given at each visit. The up-titration of the dose is completed every 4-weeks with one dose only. When the serving sized dose is reached and tolerated, the food can be maintained in the diet.

## Background

Food Protein-Induced Enterocolitis Syndrome, also known as FPIES is a non-Immunoglobulin E (IgE) mediated food allergy with reactions ranging from mild to severe shock [1]. FPIES commonly presents in infancy with symptoms of repetitive emesis starting within 1-to-4 h after ingestion. This can be accompanied by lethargy, pallor, diarrhea, abdominal distention and in its severe form, dehydration, hypotension, metabolic derangements and/or shock [1]. A chronic form of FPIES has also been described with ongoing exposure to trigger foods leading to ongoing emesis, diarrhea and failure to thrive [2]. Cow’s milk, soy and grains are the commonly reported FPIES triggers, although there are variations noted based on geographic locations [2, 3]. Although previously thought to only affect infants and children, emerging studies have described FPIES in adults which appears to be most commonly related to crustaceans [4]. The mechanism of FPIES is not well understood. It is not known whether it is truly protein that causes the resultant symptoms.

FPIES is primarily a clinical diagnosis necessitating a thorough clinical history revealing repeated reactions to the same food triggers with typical signs and symptoms, improvement upon removal of the suspected trigger and exclusion of other causes [3]. Oral food challenges (OFCs) are the gold standard for confirmation of FPIES, but reactions to the OFCs can be severe with 15% presenting with hypotension and shock and 45–95% requiring treatment with IV fluids, steroids or both [5].

Numerous protocols for OFCs in FPIES have been published, all of which need close supervision and availability of peripheral IV access and IV fluids [1]. Current guidelines for OFCs advise ingestion of a food protein dose of 0.06–0.6 g/kg in 3 equivalent doses administered over a 30-to-60-min period with doses given every 15-to-30 min. Should there be no symptoms after 2-to-3 h, a full age-appropriate food serving is then given with monitoring for another 4 h afterward. Our clinic has been performing a more cautious approach with very small up-dosing with intervals as noted in Table 1. Our approach performs up-dosing only once per month with maintenance of the tolerated OFC dose in the diet until next up-dosing. This allows us to monitor for delayed or chronic FPIES. We’ve elected to use this approach due to safety and decreased necessity for pre-emptive IV access, easy to remember up-dosing intervals and pragmatic target dosing with the final target being an estimated serving amount for the patient.

**Table 1:** Suggested modified up-dosing protocol in OFCs for the diagnosis of FPIES

Dose number	Challenge dosing of trigger food protein	Up-dosing time increments
DOSE 1	1% serving amount	4-weeks
DOSE 2	5% serving amount	4-weeks
DOSE 3	10% serving amount	4-weeks
DOSE 4	20% serving amount	4-weeks
DOSE 5	30% serving amount	4-weeks
DOSE 6	40% serving amount	4-weeks
DOSE 7	60% serving amount	4-weeks
DOSE 8	80% serving amount	4-weeks
DOSE 9	100% serving amount	4-weeks

The aim of this paper is to propose a modified approach to OFCs that allows for safer food challenges and enables monitoring for delayed or chronic FPIES reactions.

## Case presentation

The patient was first assessed at 6-months of age with an uncomplicated early infancy. She has no past medical history, no history of eczema and no active medications. Her family history is significant for atopy in her father as well as maternal and paternal grandparents. She was referred with a history of food reactions to egg.

The patient had her reaction at 6 months of age upon first ingestion of baked egg. She developed severe emesis 2 h after ingestion lasting 4 h with associated lethargy. There were no skin, respiratory, or cardiovascular symptoms. She was taken to the ER but unfortunately was not assessed quickly after her being seen by triage and so they left the ER. Her skin test was mildly positive to egg at 4 mm. She continued to avoid eggs until 17 months of age. On reassessment at 17 months of age, a repeat skin test to real egg was borderline positive at 3 mm. Our plan was to start a baked egg oral challenge following our FPIES OFC clinic protocol with dosing of 1% of the estimated serving amount on the first visit, then 5% on the second visit 4 weeks later, followed by 10% on the third visit 4 weeks later, and up-dosing as per clinic protocol in 4-week intervals. An initial OFC of 1 cc of muffin (approximately 1% serving amount) was well tolerated. She was monitored in the clinic for over 2 h. She returned home maintaining this same dose of the baked egg product in her diet daily. Within 1-week she began to experience FPIES symptoms with several episodes of watery diarrhea and two episodes of severe emesis within a 2–3 h of baked egg ingestion. Both episodes of emesis improved with ondansetron administration, but one occasion was severe enough to necessitate an Emergency Department visit. She did not require intravenous fluids in the ER. There were never signs or symptoms to suggest an IgE-mediated reaction such as skin, cardiac, respiratory symptoms or hemodynamic changes.

## Discussion

FPIES commonly presents in infancy and is usually diagnosed by a clinical history. The most common triggers in children are cow’s milk, soy and grains. OFCs are the gold standard to confirm the diagnosis of FPIES or if a food trigger has not been identified. There have been reports of very severe OFC reactions branding OFCs as high-risk and requiring these to be done in a controlled environment with readily available IV access, IV fluids, and resuscitation facilities.

Current guidelines for OFCs advise ingestion of a food protein dose of 0.06–0.6 g/kg in 3 equivalent doses administered over a 30-to-60-min period with doses given every 15-to-30 min. A full age-appropriate serving is then given if no symptoms develop within 2-to-3 h, with monitoring for another 4 h afterward [1]. In the guidelines document, we could not find the authors’ reasoning for the above dosing regimen. The authors propose protein-based dosing. We do not understand the rationale for protein based dosing, as we are not certain if protein is the cause or trigger in this condition. Diagnosis requires meeting of the major criteria as well as ≥ 3 minor criteria as follows: Major—vomiting 1-to-4 h after ingestion of the suspected trigger food with no classic IgE-mediated skin or respiratory allergy symptoms. Minor—(1) Second (or more) similar episode of emesis after ingesting the same suspected food, (2) Similar episode of repetitive vomiting 1-to-4 h after ingestion another food, (3) Extreme lethargy alongside reaction, (4) Diarrhea within 24 h, (5) Hypotension, (6) Hypothermia, (7) Need for IV fluid support, (8) Emergency Department visit due to reaction [1].

The safety concerns with the guideline’s approach to FPIES OFCs are the greater possibility of hypotension, metabolic derangements, and shock necessitating resuscitation. Per the diagnostic criteria, vomiting typically occurs 1-to 4-h after food ingestion and diarrhea may take up to 24 h to develop. With the current OFC recommendations, administering three food challenge doses in a 30-to-60-min period does not allow for this 1-to-4-h window of symptoms emergence and could result in extremely severe acute reactions due to accumulated food dosing challenges within a short period of time. Moreover, a lack of symptoms in 2-to-3 h does not mean that the food has been tolerated, as there are reactions reported up to 4 h after food ingestion. As such, providing a full age-appropriate food serving at this time poses a significant risk of a severe life-threatening reactions. In addition, assessment and monitoring of delayed FPIES symptoms must be taken into consideration with OFCs and the patients may have severe symptoms upon returning home.

As illustrated in the above case with the OFC to egg, a low dose challenge may be initially tolerated with symptoms developing days later. Although the guidelines OFC protocol is accepted by many experts in food allergy, we believe our case illustrates that this approach to OFCs should be revisited. If we had utilized the OFC method published in the guidelines, our patient would have received three challenge doses of baked egg in a 30-to-60-min period as well as an age-appropriate full dose at 2 h. We believe this would have likely resulted in a very severe and potentially dangerous reaction likely necessitating IV resuscitation. Also, we have hesitation recommending OFCs where prophylactic IV access is required as this suggests an unfavorable risk to benefit profile for the patients. Although we up-dose in a hospital clinic for severe FPIES cases, many allergists will perform OFCs in community outpatient clinics where prophylactic IV access may not be readily available. Additionally, as displayed with the egg OFC reactions occurring 1-week later, our approach allows for identification of chronic or delayed FPIES. Although our more cautious method has an increased safety profile, drawbacks include a longer time commitment of several months requiring more clinic appointments as well as the possibility of reactions occurring in the home environment.

Our proposed OFC protocol poses less risk, is easy to remember and implement with simple up dosing values and is more practical with the final target dose being an expected serving amount for the individual patient. Our proposed OFC for FPIES is to challenge patients with 1/100th of the overall target serving amount of the suspected food trigger. They should then be monitored for up to 4 h in a controlled environment with no repeat doses given to ensure true tolerability. If the challenge is tolerated, the patient will continue to ingest the same dosing at home while monitoring for symptoms of FPIES. In 4-week increments, the food challenge dosing can be increased to 5% of the serving amount, followed by 10% of the serving amount, and monthly up dosing as outlined in Table 1. We have used this protocol in over 20 patients and have never required IV access for patient resuscitation. In our experience, reactions that occur are mild and occur early in the protocol. The diagnostic criterion for a positive OFC remains. As even our conservative proposed OFC approach for FPIES may lead to severe reactions, we suggest up-dosing be performed in a hospital-based setting for patients with a severe FPIES history or in a clinic setting with availability of resuscitation equipment including intravenous access, intravenous fluids, ondansetron, dedicated and well-trained staff to facilitate resuscitation.

## Conclusions

Based on the available information, we recommend the protocol as described in the discussion and as outlined in Table 1.

Our proposed protocol allows sufficient time for FPIES symptom development with a lower initial dose of a trigger food challenge and cautious up-dosing in more prolonged 4-week increments. We believe that OFCs should be completed for confirmation of FPIES. However, we believe that the current proposed guidelines OFC will more frequently lead to severe and potentially life-threatening reactions. We propose a modified conservative protocol to allow for safer OFCs for patients. We believe our method is less likely to result in severe adverse reactions and enables monitoring for chronic FPIES. Prospective studies should be completed to assess different approaches to OFC in FPIES.

## Data Availability

Not applicable.
